# Longitudinal transcriptional analysis of peripheral blood leukocytes in COVID-19 convalescent donors

**DOI:** 10.1186/s12967-022-03751-7

**Published:** 2022-12-12

**Authors:** Mallikarjuna R. Gedda, Patrick Danaher, Lipei Shao, Martin Ongkeko, Leonard Chen, Anh Dinh, Mame Thioye Sall, Opal L. Reddy, Christina Bailey, Amy Wahba, Inna Dzekunova, Robert Somerville, Valeria De Giorgi, Ping Jin, Kamille West, Sandhya R. Panch, David F. Stroncek

**Affiliations:** 1 Center for Cellular Engineering, Department of Transfusion Medicine, National Institutes of Health, Bethesda, MD 20892 USA; 2 Section of Retinal Ganglion Cell Biology, Laboratory of Retinal Cell and Molecular Biology, National Eye Institute, National Institutes of Health, Bethesda, MD 20892 USA; 3 NanoString Technologies, Seattle, WA 98109 USA; 4 Blood Services Section, Department of Transfusion Medicine, National Institutes of Health, Bethesda, MD 20892 USA; 5 Department of Medicine (Hematology Division), University of Washington/Fred Hutchinson Cancer Center, Seattle, WA 98109 USA; 6 Infectious Disease Section, Department of Transfusion Medicine, National Institutes of Health, Bethesda, MD 20892 USA

**Keywords:** SARS-CoV2, COVID convalescent donors, Immune response, Immune exhaustion, Transcriptome, Inflammation, Cytokines

## Abstract

**Background:**

SARS-CoV2 can induce a strong host immune response. Many studies have evaluated antibody response following SARS-CoV2 infections. This study investigated the immune response and T cell receptor diversity in people who had recovered from SARS-CoV2 infection (COVID-19).

**Methods:**

Using the nCounter platform, we compared transcriptomic profiles of 162 COVID-19 convalescent donors (CCD) and 40 healthy donors (HD). 69 of the 162 CCDs had two or more time points sampled.

**Results:**

After eliminating the effects of demographic factors, we found extensive differential gene expression up to 241 days into the convalescent period. The differentially expressed genes were involved in several pathways, including virus-host interaction, interleukin and JAK-STAT signaling, T-cell co-stimulation, and immune exhaustion. A subset of 21 CCD samples was found to be highly “perturbed,” characterized by overexpression of PLAU, IL1B, NFKB1, PLEK, LCP2, IRF3, MTOR, IL18BP, RACK1, TGFB1, and others. In addition, one of the clusters, P1 (n = 8) CCD samples, showed enhanced TCR diversity in 7 VJ pairs (TRAV9.1_TCRVA_014.1, TRBV6.8_TCRVB_016.1, TRAV7_TCRVA_008.1, TRGV9_ENST00000444775.1, TRAV18_TCRVA_026.1, TRGV4_ENST00000390345.1, TRAV11_TCRVA_017.1). Multiplexed cytokine analysis revealed anomalies in SCF, SCGF-b, and MCP-1 expression in this subset.

**Conclusions:**

Persistent alterations in inflammatory pathways and T-cell activation/exhaustion markers for months after active infection may help shed light on the pathophysiology of a prolonged post-viral syndrome observed following recovery from COVID-19 infection. Future studies may inform the ability to identify druggable targets involving these pathways to mitigate the long-term effects of COVID-19 infection.

*Trial Registration*: https://clinicaltrials.gov/ct2/show/NCT04360278 Registered April 24, 2020.

**Supplementary Information:**

The online version contains supplementary material available at 10.1186/s12967-022-03751-7.

## Background

Respiratory viral infections are associated with a robust immune response. Initial activation of the innate immune response leads to the release of cytokines and chemokines. Subsequent activation of the adaptive immune response results in the production of cytotoxic T-cells directed toward virus-infected cells and B-cells that produce pathogen virus-antibodies. Following the resolution of the infection, virus-specific antibodies and cytotoxic T-cells persist, but the acute immune response resolves within days or weeks after the virus is cleared [1–3]. However, for chronic viral infections, the immune response persists, and T-cells can develop an exhausted phenotype.

Individuals infected with severe acute respiratory syndrome coronavirus-2 (SARS-CoV-2) often experience severe respiratory complications and other sequelae. SARS-CoV-2 infection results in dysregulation of the innate and adaptive immune response [4, 5]. Acute infection is associated with T-cell depletion and exhaustion, which contributes to SARS-CoV-2 persistence. More severe clinical disease is associated with greater lymphopenia [6], and recovery of lymphocyte counts precedes clinical recovery [4]. Compared to other respiratory viral infections, the immune response to SARS-CoV-2 is characterized by robust production of proinflammatory cytokines but diminished interferon Type I and III responses [7, 8]. Molecular and cellular immune features of 31 patients aged > 70 years with severe COVID-19 pneumonia have suggested that inflammation, coupled with the inability to have a proper anti-viral response, could aggravate disease severity and the worst clinical outcome [9]. Comparative host transcriptome analysis across distant coronavirus genres showed 23 pathways and 21 Differentially expressed genes (DEGs) across ten immune response-associated pathways were shared by these viruses, and these DEGs could be utilized as specific targets for novel coronavirus treatments [10].

Studies involving the convalescent period following acute viral/bacterial infections offer significant insights into disease pathophysiology, duration of immunity, host characteristics facilitating recovery, as well as susceptibility for recurrence/reinfection. In a prospective study evaluating transcriptomics of 1610 healthy subjects, 142 of whom developed an acute viral respiratory illness (influenza A, B, rhinovirus, or other) over a 2-year period, the infective phase (days 1–2) demonstrated a spike in interferon and innate immunity pathways, followed by a recovery phase characterized by transcripts implicated in cell proliferation and repair (days 4–6). By day 21, gene expression was indistinguishable from baseline in this study [1]. In another study of patients who had recovered from Ebola virus infections, a small panel of genes identified via transcriptomics were predictors of outcomes and survival, independent of viral load [11]. In a third study of convalescence, global transcriptome analysis identified diagnostic signatures for resolution and symptom persistence in Lyme disease [12].

The post convalescent period of SARS-CoV2 infections is an area of active interest. In a recent cohort study of COVID-19 infected subjects, 90% of whom had mild illness or were asymptomatic, 30% eventually reported symptoms such as fatigue, loss of taste or smell or brain fog, and an overall decrease in health-related quality of life measures up to 6 months after the acute phase [13]. Several groups have investigated the course of the antibody response of patients recovering from SARS-CoV-2 infections [14], but little is known about the recovery of transcriptomic changes in this rather protracted post-acute period in large cohorts.

We profiled peripheral blood leukocyte gene expression in people who had been infected with SARS-CoV-2 and who had recovered and were donating COVID-19 convalescent plasma. Gene expression was analyzed using the nCounter platform, a robust tool to detect the expression of 800 genes in a single reaction with high sensitivity and linearity across a broad range of expression levels. This methodology bridges the gap between genome-wide (microarrays or RNA sequencing) and targeted (real-time quantitative PCR) expression profiling [15]. Gene signatures identified on this platform have demonstrated clinical applicability in diagnostics [16] and in understanding and predicting responses to therapeutic interventions [17, 18]. Recently, the platform was utilized successfully to risk-stratify patients with active COVID-19 infections based on data from a small study [19]. We sought to investigate the transcriptome of peripheral blood post-COVID-19 in the context of other demographic, clinical, and laboratory parameters. In this study, we evaluated the immune response in COVID-19 convalescent donors (CCD). Towards this goal, using the nCounter, we analyzed and compared the transcriptomes of 162 CCD and healthy donors (HD).

## Methods

### Human subjects and eligibility criteria

Between April-December 2020 (i.e., before the COVID-19 vaccination), 162 CCD and 40 healthy donor controls were enrolled prospectively in an IRB-approved protocol (Clinical Trials Number: NCT04360278) and provided written informed consent to participate in the study. Of the 162 CCD subjects, 93 subjects donated blood once, while 46 donated twice, 12 thrice, 6 four times, and 5 donated five times.

Eligibility criteria for CCD included (1) routine blood donor criteria, (2) molecular or serologic laboratory evidence of past COVID-19 infection, and (3) complete recovery from COVID-19, with no symptoms other than residual loss of taste or smell for ≥ 28 days, or ≥ 14 days with a negative molecular test after recovery and was considered as the first visit post convalescence. We collected donor demographic and biometric data, including age, race, sex, ABO blood type, body mass index, and complete blood counts at the first visit for each subject in the early convalescent period. For each CCD, clinical severity of past COVID-19 infection was categorized as asymptomatic, mild (self-limiting course, symptomatic management at home), moderate (emergency room management or hospitalization), or severe (ICU admission). In all cases, anti-SARS-CoV-2 testing was performed. The minimum interval between plasma donations was 28 days; shorter intervals were acceptable between sample draw visits. Routine plasma donor testing was performed, including standard infectious disease testing, blood group assessment, and human leukocyte antigen antibody testing in female donors. Healthy donor control samples were obtained from research donors (protocol 99-CC-0168) who previously provided consent for the collection of research blood samples and had self-reported to be negative for SARS-CoV-2 exposure.

Anti-SARS-CoV-2 testing was performed using the Ortho-Clinical Diagnostics VITROS® Total (IgA/G/M) and IgG COVID-19 Antibody tests, as well as the SARS-CoV-2 neutralizing assay (NIH/National Institute of Allergy and Infectious Diseases (NIAID) Integrated Research Facility at Fort Detrick, Maryland, USA) as previously described [20].

### RNA isolation

Five to ten milliliters of human whole blood samples were collected in EDTA-anticoagulated tubes (BD) and centrifuged at 2500 RPM for 15 min. The supernatant plasma was separated for the antibody and multiplex immunoassays. ACK lysis buffer (Quality Biological) was added to the leftover pellet in a 1: 9 concentration, mixed several times, and incubated at room temperature for 15 min. Subsequently, the tubes were centrifuged at 1500 RPM for 10 min, and the supernatant was discarded. The pellet was washed twice with 1XPBS (KD Medical). 700 µL QIAzol lysis reagent (Qiagen) was added to the pellet with mixing and stored at −80 °C. Using the RNeasy Mini Kit (Qiagen), RNA was eluted in 40 µL of Milli-Q water. Following quality (Agilent 2100 Bioanalyzer) and quantity (Nanodrop One, Thermo Scientific) checks, the RNA was stored at −80 °C for further transcriptomic profiling.

### Nanostring nCounter transcriptomic profiling

Nanostring transcriptomic profiling was performed using the nCounter^®^ Human Host Response (Additional file 1: Table S1) and the nCounter^®^ Human TCR diversity panels (Additional file 2: Table S2). Whole blood total RNA (100 ng) was hybridized to reporter and capture probes at 65 °C for 16 h using a thermal cycler (Veriti Applied Biosystems). These hybridized samples were loaded onto the nCounter cartridge, and the post hybridization step and scanning were performed on the nCounter Prep Station and Digital Analyzer.

### Multiplex immunoassay

In a subset of CCD samples with highly perturbed gene expression and in healthy donor controls, we performed cytokine analysis. According to the manufacturer's instructions, a multiplex biometric immunoassay was performed to assess 48 cytokine and chemokine cell signaling molecules (Bio-Plex Human Cytokine Assay; Bio-Rad Inc., Hercules, CA, USA) [21]. The quantified cytokines included interleukins (IL-1α, IL-1β, IL-1Ra, IL-2, IL-2Rα, IL-3, IL-4, IL-5, IL-6, IL-7, IL-8, IL-9, IL-10, IL-12p40, IL-12p70, IL-13, IL-15, IL-16, IL-17A, & IL-18), interferons (IFN-α2 & IFN-γ), tumor necrosis factors (TNF-α, TNF-β, & TRAIL), growth factors (SCF, FGF, β-NGF, HGF, LIF, PDGF-BB, VEGF, SCGF- β, G-CSF, M-CSF, & GM-CSF), and chemokines (CCL247CTACK, eotaxin, GRO-α, CXCL10/IP-10, CCL2/MCP-1, CCL7/MCP-3, MIF, MIGCCL3/MIP-1α, CCL4/MIP-1β, CCL5/RANTES, & SDF-1α). A multiplex array reader from Luminex™ Instrumentation System (Bio-Plex 200 system) was used to determine the cytokine levels. The Bio-Plex Manager Software was used to calculate the cytokine concentrations.

### Data processing for nCounter host response panel

All statistical analyses were performed using R (Version 4.1.1). Raw counts were normalized by scaling each sample by its geometric mean of the panel’s 12 housekeeping genes. Of the 270 samples from CCDs, we removed 2 samples with low signal strength, defined as low outlier values of the housekeeper geometric mean. The normalized data were then log2-transformed. Healthy donor samples were compared to CCD across 4 time windows: 26–89 days, 90–119 days, 120–149 days, and 150–241 days post-symptoms-onset. Within each window, a linear mixed model was fit to each gene’s normalized log2-transformed expression. The model treated CCD/healthy donor status, age, sex, and race as fixed effects and patient ID as a random effect. No patients had multiple samples within the 120–149 day window, so in this window, a linear model was fit with no random effects. The R library lmerTest was used to fit mixed models, and the R function lm was used to fit linear models. For each window, all genes’ p-values were converted to False Discovery Rates using the Benjamini–Hochberg procedure, using the R function p.adjust.

### Classification of highly perturbed samples

To calculate perturbation scores, we began by standardizing the data to give each gene mean 0 and standard deviation 1 within the healthy donor samples. We then defined a perturbation score as each sample’s Euclidean distance from the mean healthy donor sample in this standardized expression data. To define “highly perturbed” samples, we used the R library Mclust to cluster perturbation scores into two clusters, one high and one low. The highly perturbed samples were clustered into groups P1 and P2 by applying the R functions hclust and cutree to their log2-transformed normalized expression data.

### Analysis of nCounter TCR diversity panel

TCR diversity scores were calculated using the Rosalind nCounter TCR Diversity Report, a software tool designed specifically for the nCounter TCR diversity panel. The software calculates the Shannon diversity index for each sample’s TCR gene counts. Gene expression values are first normalized to a “panel standard” reference sample to remove variability due to batch effects. TCR diversity scores were analyzed using the same statistical models applied to gene expression values.

## Results

### Demographics and clinical characteristics

Whole blood samples were collected from 270 donations by CCD and 40 contemporaneously recruited healthy donors from April to December 2020. The 270 CCD donations were consecutive, and they were from 162 donors. Among the 162 CCD, 93 donated once, and 69 donated more than once. All 40 healthy donors only donated once.

Age, sex, ethnicity, and Body Mass Index (BMI) distributions were similar among the 162 CCD and 40 healthy donors (Table 1). With respect to baseline complete blood counts, hemoglobin, platelets, and absolute basophil and eosinophil counts were similar among CCD and healthy donors. However, despite sample collections occurring over several months after convalescence, mean counts for absolute neutrophil counts (ANC), absolute monocyte counts (AMC), and absolute lymphocyte counts (ALC) were significantly higher among CCD compared to healthy donors (Table 1). Cell counts were collected earlier post convalescence for CCD only with their first donation. Among the CCDs, most had mild disease, and anti-SARS-CoV2 levels were highly variable. Some CCDs donated up to 5 times (Table 2).

**Table 1:** COVID-19 Convalescent Donor Demographics and Blood Counts

	CCD (n = 162)	HD (n = 40)	P Value
Sex (n, %)			
Male	75 (46.6)	23 (57.5)	–
Female	87 (53.3)	17 (42.5)	–
Age (years)	49.3 ± 6.2	50.9 ± 14.5	ns
Ethnicity (n, %)			
Caucasian	122 (75.3)	25 (62.5)	–
African American	13 (8.0)	7 (17.5)	–
Asian	11 (6.7)	5 (12.5)	–
Hispanic	7 (4.3)	2 (5)	–
Mixed	4 (1.2)		–
Others	2 (0.6)	1 (2.5)	–
Declined	2 (0.6)	-	–
Unknown	1 (0.3)	-	–
BMI	27.7 ± 6.2		
Cell counts at baseline#			
WBC (4.5–11.0 × 10^9^/L)	6.2 ± 1.4	5.1 ± 1.4	0.0017
Hemoglobin (13.2–16.6 gm/dL)	13.7 ± 1.21	13.7 ± 1.6	ns
Platelets (150–400 × 10^9^/L)	231.8 ± 49.9	231.2 ± 54.2	ns
Neutrophil Absolute (ANC) (2.5–7.5 × 10^9^/L)	3.6 ± 1.1	2.8 ± 0.8	0.0315
Lymphocytes Absolute (ALC) (1.0–4.0 × 10^9^/L)	1.8 ± 0.5	1.3 ± 0.2	0.0103
Monocytes Absolute (AMC) (0.2–0.8 × 10^9^/L)	0.51 ± 0.12	0.34 ± 0.08	< 0.0001
Eosinophils Absolute (0–0.5 × 10^9^/L)	0.15 ± 0.11	0.20 ± 0.07	ns
Basophils Absolute (0.00–0.30 × 10^9^/L)	0.04 ± 0.02	0.04 ± 0.01	ns

*CCD* COVID-19 convalescent plasma donors, *HD* Healthy Donors

^#^Cell counts were obtained only at the first donation for repeat donors

**Table 2:** Number of Donations by COVID-19 Convalescent Plasma Donors and Clinical Characteristics of Their Infections

	CCDs (n = 162)
Donations (n, %, days post symptom onset)	270 (100), 116.3 ± 43.9
Single	93 (34.4), 108.8 ± 44.8
Twice	46 (34.8), 120 ± 40.9
Thrice	12 (14.4), 126.6 ± 46.6
Four	6 (8.8), 113.2 ± 42.1
Five	5 (7.4), 121.7 ± 48.6
Disease severity (n, %)	
Mild	148 (91.4)
Moderate	10 (6.1)
Severe	3 (1.8)
Asymptomatic	1 (0.6)
Blood Group (n, %)	
O	67 (41.1)
A	57 (36.8)
B	22 (13.4)
AB	11 (6.7)
Unknown	5 (3.0)
Antibodies	
Total Anti-SARS-CoV-2	392.6 ± 300.2
IgG Anti-SARS-CoV-2	10.66 ± 7.59
Neutralizing Antibody (n, %)	270 (100)
< 1:40	9 (3.3)
1:40	83 (30.7)
1:80	65 (24.0)
1:160	28 (10.3)
1:320	9 (3.3)
1:640	2 (0.7)
None	72 (26.6)
Not Tested	1 (0.3)
Pending	1 (0.3)

*CCD* COVID-19 convalescent plasma donors, *HD* Healthy Donors

^*^

Age, ethnicity, sex, BMI, and baseline cell counts, as well as ABO blood groups, disease severity and mean total and IgG antibodies, and median neutralizing antibody titers are summarized for CCD donating multiple times (Table 3). Serial antibody titers of individuals who donated 1, 2, 3, 4, or 5 times are shown in Additional file 5: Fig. S1, A-C. The total mean antibody levels increased at the last donation compared to the first donation [Signal/Cutoff (S/Co) 495.0 ± 315.3 versus 376.6 ± 276.4, p < 0.0001]. Otherwise, no differences were observed in this cohort of individuals with respect to changes in antibody titers over collections or when evaluated by other parameters.

**Table 3:** COVID-19 Convalescent Plasma Donor Demographics Based on Number of Donations

	Single	Double	Three	Four	Five	P Value
Number	93	46	12	6	5	
Age (years)	46.77 ± 15.32	48.5 ± 15.32	54.47 ± 14.03	47.5 ± 15.82	55.68 ± 6.65	0.0143
Sex (n, %)						
Male	46 (49.4)	18 (40.4)	5 (46.1)	2 (33.3)	4 (75)	
Female	47 (50.5)	28 (59.5)	7 (53.8)	4 (66.6)	1 (25)	
Ethnicity (n, %)						
Caucasian	75 (80.6)	31 (68.0)	10 (84.6)	2 (33.3)	4 (75)	
African American	9 (9.6)	4 (8.5)				
Asian	3 (3.2)	7 (14.8)		1 (16.6)		
Hispanic	1 (1.07)	2 (4.2)	2 (18.1)	1 (16.6)	1 (25)	
Mixed	2 (2.1)	1 (2.1)		1 (16.6)		
Others		1 (2.12)		1 (16.6)		
Declined	2 (2.1)					
Unknown	1 (1.0)					
BMI	27.53 ± 5.91	27.39 ± 6.283	25.12 ± 3.46	27.24 ± 3.93	33.86 ± 8.16	< 0.0001
Severity (n, %)						
Mild	87 (93.5)	44 (95.7)	9 (76.9)	5 (83.3)	3 (50)	
Moderate	5 (5.3)	1 (2.1)	2 (15.3)	1 (16.6)	1 (25)	
Severe	1 (1.0)		1 (7.6)		1 (25)	
Asymptomatic		1 (2.12)				
Anti-SARS-Cov2						
Total Anti-SARS-CoV-2	291.9 ± 262.4	403.5 ± 288	544.4 ± 299.4	372.5 ± 330.6	527.4 ± 321.4	< 0.0001
IgG Anti-SARS-CoV-2	8.148 ± 6.517	10.42 ± 7.574	14.31 ± 8.038	10.3 ± 8.265	15.92 ± 6.792	< 0.0001
Median Neutralizing Abs	1:40	1:80	1:80	1:80	1:80	

*CCD* COVID-19 convalescent plasma donors, *HD* Healthy Donors

### Gene expression remains altered for months after infection

CCD samples were stratified into 4 groups based on time since symptoms onset: 26–89 days, 90–119 days, 120–149 days, and 150–241 days. For each gene within each of these groups, a linear mixed model was fit comparing log2-transformed expression levels in CCD vs. healthy donors, adjusting for age, sex, and race, and treating donor ID as a random effect. Results from these models are in Additional file 3: Table S3.

**Fig. 1:**
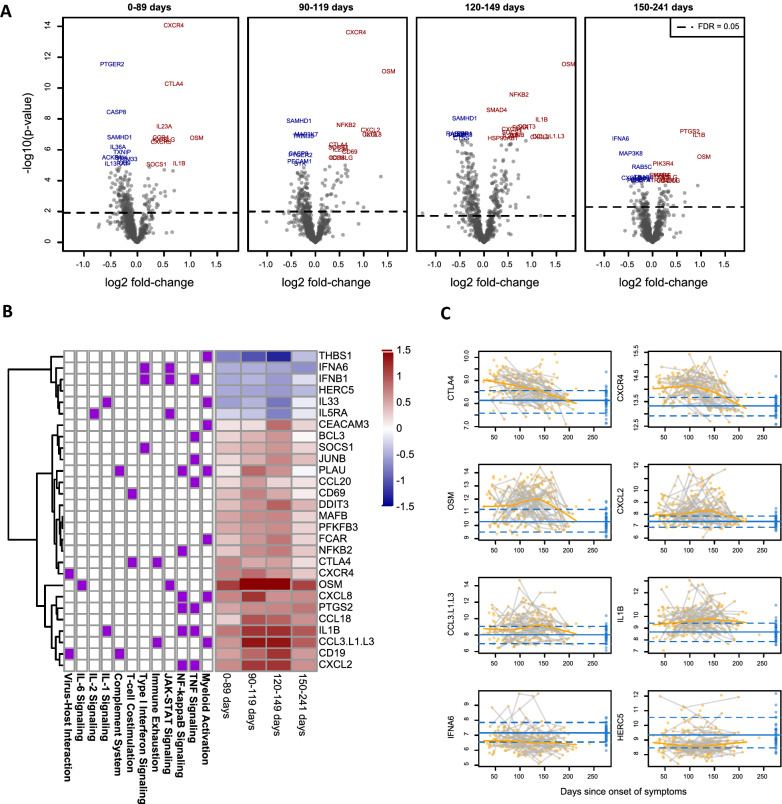
Differential expression of CCD vs. HD. **A**. Log2 fold-change and -log10(p-value) of the expression of genes in CCD vs. HD. CCD samples were partitioned into 4 groups based on days post-onset of symptoms, and each group was analyzed separately. **B**. Expression of all genes achieving a fold change of > 50% and a False Discovery Rate < 0.05, estimated log2 fold-change vs. HD by time window. Side bars show gene set membership. **C**. Expression of selected genes by time window. Point color shows CCD (orange) vs. HD (blue), and blue lines show the mean and 0.1/0.9 quantiles of HD expression

All CCD time windows saw multiple genes with highly statistically significant changes from healthy donors (Fig. 1A). From the panel’s 775 non-housekeeper genes, a fold-change of > 20% and a False Discovery Rate < 0.05 were found for 85 genes in the 26–89-day window (n = 77), 87 genes in the 90–119-day window (n = 56), 178 genes in the 120–149-day window (n = 60), and 30 genes in the 150–241-day window (n = 58).

28 genes departed from the healthy donor mean by > 50% and with FDR < 0.05 in at least one time window (Fig. 1B). These genes participate in diverse biological pathways. While some genes gradually but monotonically return to healthy donor levels, others see their greatest departures from healthy donor expression 120–149 days after onset of symptoms (Fig. 1C). The genes with the greatest average departures from healthy donors include *CTLA4*, *CXCR4*, *OSM*, *CXCL2*, *CCL3/CCL3L1/CCL3L3*, *IFNA6*, and *HERC5*. *CTLA4* and *CXCR4* begin upregulated above healthy donor medians and gradually return to normal (Fig. 1C).

### Two clusters of CCD samples with “highly perturbed” gene expression demonstrate aberrant cytokine expression

**Fig. 2:**
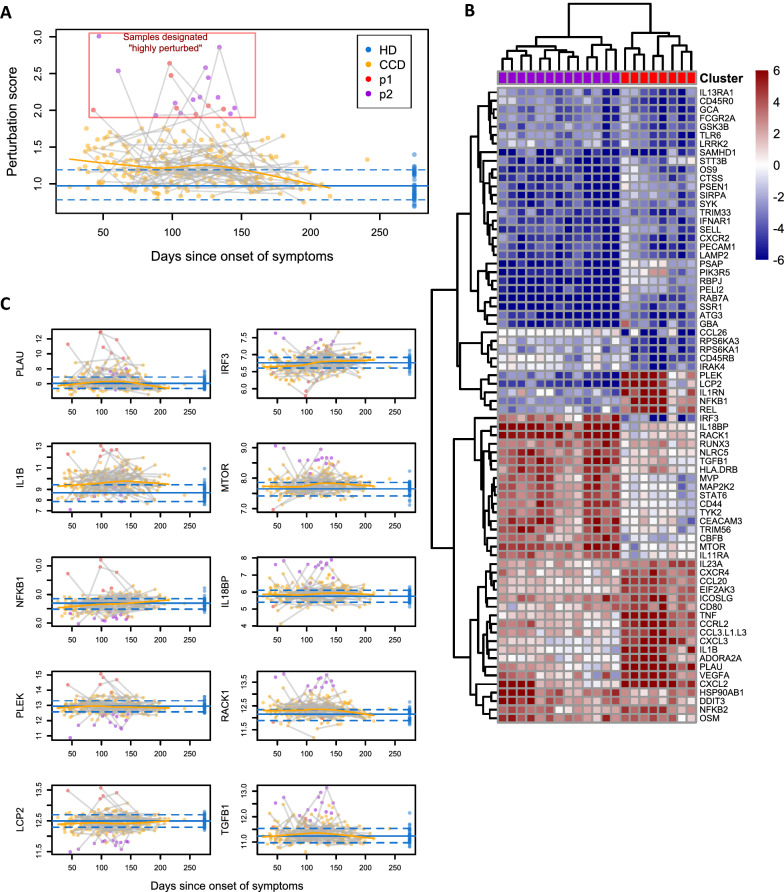
Subset of samples with highly perturbed expression profiles. **A**. Perturbation scores plotted over time. Point color shows CCD/HD status and clustering results from 21 highly perturbed samples. Blue lines show the mean and 0.1/0.9 quantiles of HD samples. **B**. Gene expression in highly perturbed samples, standardized to the mean and SD of the HD samples. The 20 most upregulated and down-regulated genes in each cluster are shown. **C**. Expression of genes characteristic of P1 and P2. Blue lines show the mean and 0.1/0.9 quantiles of HD expression

In efforts to further study these persistent or recurring and prolonged gene expression aberrations, we sought to delineate samples with immune states perturbed far beyond the average trend. We defined a perturbation score based on the Euclidean distance of each sample’s expression profile from the mean healthy donor sample (Methods). Average CCD perturbation scores were elevated at early time points and returned to the mean healthy donor levels by 200 days (Fig. 2A). CCD perturbation scores were highly right tailed, with a subset of samples from ~ 150 days post-symptoms onset falling far above the healthy donor range. Model-based clustering partitioned the perturbation scores into a large group of “typical” samples and a group of 21 “highly perturbed” CCD samples. This study cannot definitively attribute these highly perturbed immune states to earlier COVID-19; however, detailed donor history and the need for complete absence of symptoms during repeat donations preclude the possibility of re-infections as cause for these changes. Additionally, the study period (carried out early in the pandemic) ruled out the possibility of vaccine induced changes. With this caveat, the below results may offer clues to COVID-convalescent immune dysregulation.

The 21 highly perturbed samples fell into 2 distinct clusters based on their expression profiles. Cluster “P1” (8 samples) was characterized by high expression of *PLAU, IL1B, NFKB1, PLEK, LCP2*, and other genes (Fig. 2B, C). In a study of time-order transcriptomics to characterize molecular mechanisms which underpin multiple organ dysfunction in COVID-19, *PLAU* (plasminogen activator, urokinase) was among the genes to induce olfactory and neurological dysfunction [22]. *NFKB1*, an NF-κB signaling pathway gene, has been involved in the upregulation of inflammatory responses in patients with COVID-19 infection, with TLR4 acting as an intermediary. Additionally, despite a diminished IFN-I response, robust cytokine production and viral replication in SARS-CoV-2 infection are thought to be due to virus-mediated activation of NF-κB in the absence of other canonical IFN-I-related transcription factors [23–25]. *PLEK* and *LCP2* genes which code proteins, pleckstrin and lymphocyte cytosolic protein 2, respectively, may play a role in COVID-19 pathogenesis [26, 27].

Cluster “P2” (13 samples) was characterized by high expression of *IRF3, MTOR, IL18BP, RACK1, TGFB1* and others. TLR3 and TLR4 activate IRF3 (Interferon Regulatory Factor 3) during the viral attack, triggering the type I interferons (IFN-1) transcription and NF-κB activation through the TRIF-dependent pathway during SARS-CoV-2 [28]. This, in turn, changes the expression of many genes that trigger inflammatory and antiviral responses. In severe COVID-19, SARS-CoV-2 triggers a chronic immune reaction that is instructed by TGF-β [29]. Excessively elevated TGF-β activity is also a key feature of COVID-19 cytokine storm [30]. During SARS-CoV-2 infection and replication, mTOR, a serine-threonine kinase involved in cell proliferation and cellular metabolism, was found to be active [31]. mTOR is involved in the interaction of adapter proteins MyD88, TLR9, and IRF-7 in plasmacytoid dendritic cells (pDCs), which leads to the transcriptional activation of type-I interferon (IFN) genes. The interleukin 18 binding protein (IL18BP) gene encodes a soluble inhibitor and carrier that keeps proinflammatory cytokine IL-18, a natural killer (NK) cell amplifier in check [32, 33]. Both T-cells and NK cells both produce IFN-γ, and IL-18 plays a critical role in this process. IL-18, along with other cytokines (IFN-γ, IL-1, IL-6, TNF), are elevated in cytokine storm and are thought to have central immunopathologic roles in COVID-19 [34].

### Altered TCR diversity and aberrant cytokine expression in perturbed subset

**Fig. 3:**
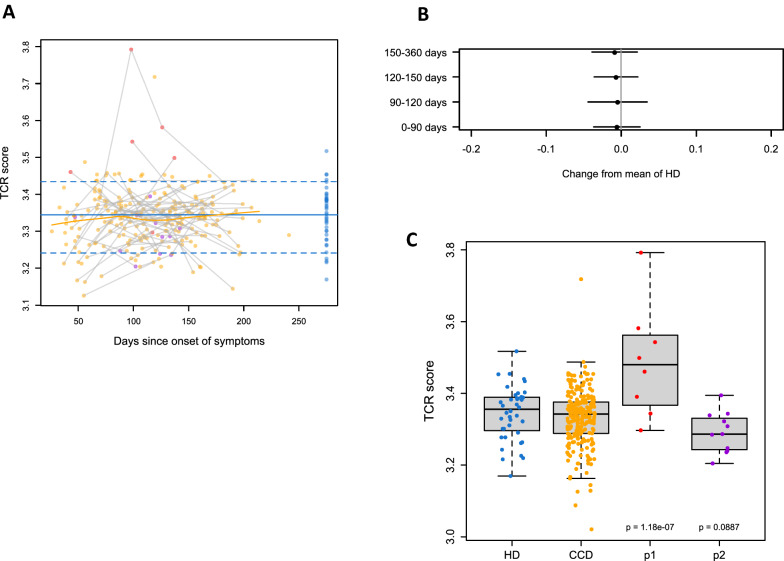
Changes in TCR diversity. **A**. TCR diversity score of all samples over time. **B**. Mean changes in TCR diversity score from HD to CCR. Points show estimates, lines show 95% confidence intervals. **C**. TCR diversity scores in HD, CCD, and perturbed CCD clusters (P1, P2). P-values contrast P1 and P2 to HD

We compared the TCR repertoires of 270 CCDs and 40 healthy donors using Nanostring's TCR diversity panel. The average TCR diversity score in CCD samples did not depart from the average healthy donor sample at any time point (Fig. 3A, B). However, the T-cell receptor (TCR) diversity score in perturbed subset P1 was significantly elevated compared to healthy donors (p = 1.18X10^−7^) (Fig. 3C), with unique T cell clonal expansion. Further analysis of the VJ gene combination revealed a significantly increased expression of 7 VJ pairs (*TRAV9.1_TCRVA_014.1, TRBV6.8_TCRVB_016.1, TRAV7_TCRVA_008.1, TRGV9_ENST00000444775.1, TRAV18_TCRVA_026.1, TRGV4_ENST00000390345.1, TRAV11_TCRVA_017.1*) while 54 pairs declined with FDR < 0.05 (Additional file 4: Table S4). TCR is crucial in T cell-mediated viral clearance and TCR bias is notable in various diseases [35]. Clonotypic T cell receptors (TCRs), which identify a peptide (8–15 amino acids) presented by major histocompatibility complex (MHC), direct the signaling that T cells use to orchestrate adaptive immunity [36, 37]. During the acute stages of infection, peptide-MHC complex (pMHC) recognition by TCR causes naive T cells to become activated and differentiate into diverse functional subsets, which eradicates invasive pathogens [38]. The variable (V), junctional (J), and constant (C) regions make up each of the two TCR chains (α and β) [39]. The diversity (D) region establishes an essential chain by joining the V and J areas. Thus, a functional and highly varied TCR repertoire is created by the TCR recombination process, which also develops highly diverse complementarity-determining regions (CDRs) localized in the TCR α and β chains. Luo et al*.* studied the blood T cells from recovered COVID-19 patients PBMCs from 1 to 6 weeks, and their TCR repertoires and immune metabolic processes were analyzed using single-cell TCR-seq and RNA-seq [36]. They observed that the TCR repertoire's diversity increased in patients who were discharged but it quickly went back to its baseline levels after 1 week after the SARS-CoV2 virus was eliminated. A significant shift in gene signatures from antiviral response to metabolic adaptation correlated with the dynamics of T cell repertoire in the study. By using ImmunoSEQ technology, Wang et al*.* studied the TCR repertoires of COVID-19 patients' PBMCs obtained before (baseline), during (acute), and after rehabilitation (convalescent) and they discovered that these patients TCR repertoires differed noticeably from healthy controls in terms of decreased TCR diversity, abnormal complementary-determining region 3 (CDR3) length, different TRBV/J gene usage, and higher TCR sequence overlap [40].

**Fig. 4:**
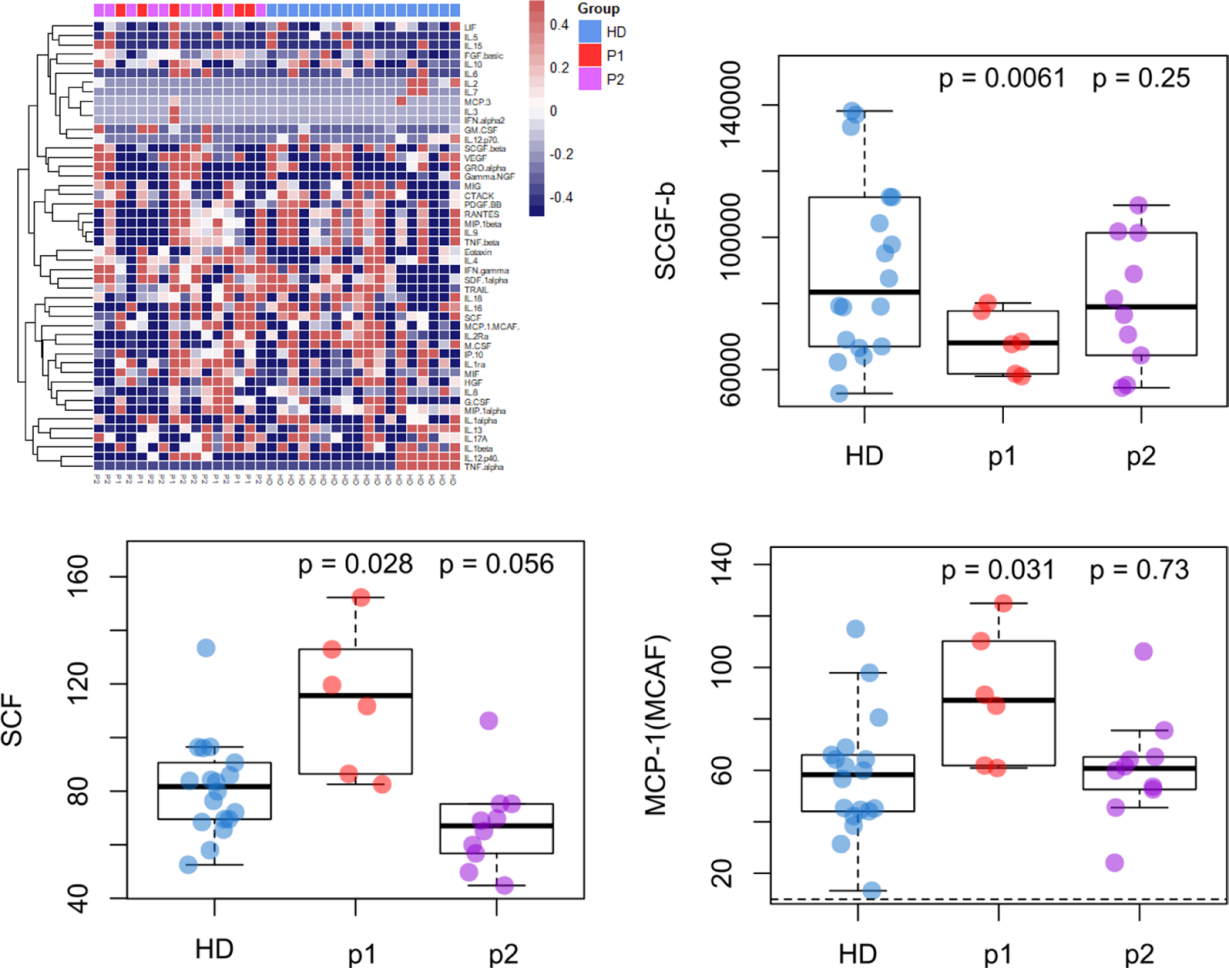
Multiplexed cytokine analysis from a limited time window comparing healthy donor (HD) vs. the highly perturbed CCD clusters (P1 and P2) identified by gene expression profiling. The 3 cytokines with statistically significant departures from HD in either P1 or P2 are shown

Multiplexed cytokine analysis was performed on 18 healthy donors, 6 P1 CCD, and 10 P2 CCD. Of the 48 cytokines analyzed, the P2 cluster had none that differed significantly from healthy donors, and the P1 cluster had 3 cytokines with significant differences from healthy donor: stem cell factor (SCF), Monocyte Chemoattractant Protein-1 (MCP-1), and Stem Cell Growth Factor-beta (SCGF-b) (Fig. 4). SCF overexpression is notable in inflammatory conditions. Binding of SCF to c-Kit leads to activation of multiple pathways, including phosphatidyl-inositol-3 (PI3)-kinase, phospholipase C (PLC)-gamma, Src kinase, Janus kinase (JAK)/Signal Transducers and Activators of Transcription (STAT) and mitogen-activated protein (MAP) kinase pathways. SCF is an important growth factor for mast cells, promoting their generation from CD34 + progenitor cells. In vitro, SCF induces mast cells survival, adhesion to extracellular matrix, and degranulation, leading to the expression and release of histamine, proinflammatory cytokines, and chemokines. SCF also induces eosinophil adhesion and activation. SCF is upregulated in inflammatory conditions both in vitro and in vivo in humans and mice [41]. MCP-1 elevation is suggestive of increased viral clearance from the CNS [42, 43]. SCGF, which was also marginally elevated in the healthy donor compared to the 2 CCD groups, may be a marker for hematopoietic recovery [44].

Demographic and laboratory data did not identify significant differences between the 2 perturbed clusters compared to healthy donor or “other” CCD samples (Table 4). We were unable to perform a look-back review of donors concerning the occurrence/persistence of signs or symptoms consistent with long-COVID syndrome in our cohort.

**Table 4:** COVID-19 Convalescent Plasma Donor Demographics Comparing P1, P2 Perturbed Sample Clusters (P1, P2) with Other CCD and Healthy Donors

	HD	P1	P2	Other CCD	P Value
Number	40	8*	13	249*	
Age (years)	50.9 ± 14.5	47.8 ± 12.9	47.3 ± 14.6	49.3 ± 14.9	0.9003
Sex (n, %)					
Male	23 (57.5)	5 (62.5)	10 (76.9)	110 (44.1)	-
Female	17 (42.5)	3 (37.5)	3 (23.1)	139 (55.8)	-
Ethnicity (n, %)					
Caucasian	25 (62.5)	5 (62.5)	10 (78.5)	180 (72.2)	-
African American	7 (17.5)			17 (6.82)	-
Asian	5 (12.5)	1 (12.5)	1 (7.69)	19 (7.63)	-
Hispanic	2 (5)		1 (7.69)	19 (7.63)	-
Mixed		2 (25)		6 (2.4)	-
Others	1 (2.5)			6 (2.4)	-
Declined	-		1 (7.1)	1 (0.4)	-
Unknown	-			1 (0.4)	-
BMI		25.1 ± 2.5	26.5 ± 5.9	27.8 ± 6.2	0.3663
Severity (n, %)					
Mild	-	7 (87.5)	11 (84.6)	219 (87.9)	
Moderate	-		2 (15.3)	20 (8)	
Severe	-			9 (3.6)	
Asymptomatic	-	1 (12.5)		1 (0.4)	
Cell counts at baseline#					
WBC	5.1 ± 1.4	6.0 ± 1.0	6.3 ± 1.9	6.2 ± 1.4	0.0186
Hemoglobin	13.7 ± 1.6	13.7 ± 0.5	14.3 ± 0.9	13.6 ± 1.2	0.3501
Platelets	231.2 ± 54.2	214.4 ± 14.8	241.4 ± 53.7	231.8 ± 50.4	0.6972
Neutrophil Absolute (ANC)	2.8 ± 0.8	3.5 ± 0.9	3.9 ± 1.8	3.6 ± 1.0	0.1345
Lymphocytes Absolute (ALC)	1.3 ± 0.2	1.7 ± 0.6	1.6 ± 0.3	1.8 ± 0.5	0.0476
Monocytes Absolute (AMC)	0.34 ± 0.08	0.5 ± 0.07	0.4 ± 0.1	0.5 ± 0.1	0.0003
Eosinophils Absolute	0.20 ± 0.07	0.14 ± 0.1	0.16 ± 0.1	0.15 ± 0.1	0.6322
Basophils Absolute	0.04 ± 0.01	0.04 ± 0.01	0.04 ± 0.02	0.04 ± 0.02	0.9957
Anti-SARS-Cov2					
Total Anti-SARS-CoV-2		164.6 ± 158.0	409.9 ± 323.4	399.1 ± 300.5	0.0917
IgG Anti-SARS-CoV-2		5.3 ± 5.2	10.9 ± 6.1	10.8 ± 7.6	0.1285

^*^Donations from same COVID-19 convalescent plasma donor at different time intervals

^#^Cell counts were obtained only at the first donation for repeat donors

## Discussion

We evaluated the transcriptome of peripheral blood leukocytes from people who had recovered from COVID-19 and donated convalescent plasma. At the time of the donation, the CCD had no COVID-19 symptoms, tested negative for SARS-CoV2, and were considered healthy because they passed a blood donor health history questionnaire. The CCD differed from healthy donors in several respects. When compared to healthy donors, CCD had significantly higher leukocyte, lymphocyte, and monocyte counts (early on in convalescence), as noted previously with other viral infections as well [45, 46]. More importantly, CCD demonstrated significant differences in peripheral blood leukocyte transcriptomes. In a subset of CCD with highly perturbed transcriptomics, cytokine levels were also abnormal in PBMC samples collected months after convalescence. These results suggest that the immune dysregulation occurring during acute infection in COVID-19 persists for several months post-infection.

Our study is unique in that we analyzed convalescent donors over a long period. Some studies have evaluated people serially with SARS-CoV-2 and found persistent changes in cellular immunity, but only studied patients for 6 to 10 weeks following resolution of COVID-19 [47, 48]. Our longitudinal assessment of PBMC samples from CCD identified unique transcriptomic trends. The CCD samples were collected at various time intervals following the diagnosis of COVID-19. The samples were collected from a few weeks to more than 6 months post-symptom resolution. While we found some differences in gene expression among CCD at all time intervals, the nature of transcriptomes varied with time. Interestingly, when compared to healthy subjects, the number of differentially expressed genes increased over time, peaked at about 120 to 150 days post-symptom resolution, and then fell during the remainder of the study period.

The function of the differentially expressed genes also changed with time. Initially, less than 90 days post-symptom resolution, genes in interferon signaling, TNF signaling, and cell exhaustion pathways were expressed at high levels in CCD. Later, as the expression of CTLA-4, an inhibitor of T-cell function and marker cell exhaustion, fell in CCD leukocytes, the expression of genes in TGF-β signalizing, TNF signaling, IL-6 signaling, and myeloid activation increased in CCD leukocytes. After 120 to 149 days post-symptom resolution, the number of differentially expressed genes fell, but the proinflammatory genes OSM, PTGS2, and IL1B remained up-regulated. The expression of immunological checkpoint inhibitor CTLA4 is enhanced on the surface of T-cells due to induction of INF-γ production by neutrophils and monocytes, which are abundant in the peripheral blood of people with COVID-19 [49]. An earlier analysis of publicly available transcriptomic databases found that the number and intensities of these inhibitory receptors were higher in SARS-CoV-2 infections compared to SARS-CoV-1, influenza, and respiratory syncytial virus infections [50]. Besides CTLA4, an increase in activated CXCR4 + T cells homing to the lungs is associated with fatal COVID-19 [51]. Hou et al. identified a significant enhancement of the expression of inhibitory receptors, which included CTLA-4 on SARS-CoV-2–specific CD4 + T cells (suggesting an exhausted phenotype) even though the quantity of SARS-CoV-2–specific CD4 + T cells in convalescent COVID-19 patients was maintained after a year of recovery [52]. In convalescent subjects with mild/moderate symptoms, 27–47 days after symptom onset, the T-cell differentiation regulation and memory T cell-related gene *CXCR4* were upregulated along with *FOS*, *JUN*, *CD69*, and *CD83* [53]. Hence, both altered *CTLA4* and *CXCR4* expression levels may play a critical role in the severity and fatality of the SARS-CoV-2 infection, as well as during convalescence.

*OSM*, *CXCL2*, and *CCL3/CCL3L1/CCL3L3* (jointly measured with a single probe) initially have wide expression ranges spanning from the normal range of healthy donor samples to greater than 16-fold increases from the healthy donor mean. By 200 days, these extreme over-expression values are no longer observed, and these genes’ mean expression returns to the healthy donor mean. The *OSM* gene encodes the protein Oncostatin M, a pleiotropic cytokine that stimulates IL-6. Circulating OSM positively correlates with COVID-19 severity. IL-6, a proinflammatory cytokine, drives immune dysregulation and respiratory failure leading to higher mortality [54, 55]. The chemokine CXCL2 is critical for macrophage, monocyte, and neutrophil migration and is also known to facilitate the clearance of SARS-CoV2 in the absence of CD4 + and CD8 + T cells or neutralizing antibodies beyond 12 days of infection [56]. The *CCL3L3* gene encodes the CCL3 protein (MIP-1), one of the chemokine families with diverse functions based on the C–C motif. CCL3 is a neutrophil chemotaxis protein that acts as a ligand for CCR1, CCR3, and CCR5. Neutrophils play a significant role in COVID-19 severity, as CCL3 is upregulated in severe COVID-19 [57] [58]. *IL1*B shows a similar pattern, but it did not return to the healthy donor average by 200 days. Interleukin (IL)-1β, a potent proinflammatory cytokine, plays a significant role in the host defense response to infection and injury. Studies have shown elevated levels of IL1β during COVID-19 infection [59]. Additionally, the IL1 family of cytokines plays a key role in inducing cytokine storm in poorly controlled COVID-19 infection. Furthermore, in a recent clinical study of 88 hospitalized subjects with SARS-CoV-2 infection, blockage of IL1β with canakinumab demonstrated better clinical outcomes [59]. *IFNA6* and *HERC5* both show consistent down-regulation from healthy donors, but with high outliers > fourfold above the healthy donor mean. These genes remain suppressed below the healthy donor average beyond 200 days. Type I interferon subtype IFNA6 has been reported in patients infected with COVID-19 in the context of platelet degranulation and B cell maturation [60–62]. E3 ligase HECT and RCC1-containing protein 5 (HERC5) regulate interferon-stimulated gene 15 (ISG15) signaling in response to SARS-CoV-2 and other viral infections [63].

A subset of “highly perturbed” CCD had more marked changes in gene expression. These gene expression changes in the perturbed CCD seemed transient. Of the 21 patients with a “highly perturbed” sample, 11 had multiple timepoints collected. Among these 11 individuals, only 1 was highly perturbed at multiple timepoints, transitioning from cluster P2 at 88 days to cluster P1 at 117 days. A subgroup of these perturbed donors had gene expression changes showing interferon production and innate immune system activation, lower levels of anti-COVID antibodies and increased TCR diversity.

It is unclear why immune changes were found in CCD up to 6-months post-symptom resolution. However, it has proven difficult to find the immunological "bridge" that connects acute COVID-19 and post-COVID-19 syndrome [64, 65]. Careful annotation of the clinical symptomatology is a crucial step in understanding the pathophysiology of the post-COVID syndrome. It may be possible to separate disease drivers by separating residual symptoms of the acute disease site from new symptoms that may develop after the acute disease recovery. Moreover, confounding factors may also include post-traumatic stress disorder (PTSD)-related elements, which can make it difficult for patients to accurately assess their own clinical symptoms and necessitate comprehensive neuropsychiatric assessments [65]. Additionally, persistence of SARS-COV-2 has been detected by RT-PCR in respiratory specimens for approximately 2 to 3 weeks post-infection, in some cases 4 to 8 weeks [66]. SARS-CoV-2 can be detected in feces for a longer period than in respiratory specimens [66, 67]. One study found that SARS-CoV-2 could be detected in respiratory samples for a median of 14 days and in feces for a median of 19 days [68]. Another study found that it could be detected in feces for 10 weeks [67]. The persistent shedding of SARS-CoV2 is not thought to be due to reinfection but is more likely the result of release of sequestered virus or mutation of the original virus. It is also possible latent virus is reactivated. However, persistent SARs-CoV2 has not been detected 6-months post-symptom resolution.

The presence of prolonged changes in immune cell transcriptomes post-COVID-19 is consistent with other studies reporting prolonged symptoms in people who have recovered from acute infections. Many people experience post-acute sequelae of COVID-19 (PASC) which is also known as long COVID or long haulers syndrome. These people experience fatigue, tiredness, dyspnea, shortness of breath, chest pain, joint pain, and perceived cognitive impairment. One study found that 93% of people hospitalized for COVID-19 experienced PASC [69]. In the same study, among people who had visited a clinic, 55% had at least one of these persistent symptoms 25 to 89 days post-diagnosis, and 67% had at least one persistent symptom 90 to 174 days post-diagnosis. After 175 days, 64% of people experienced symptoms [69]. It is possible that our study included CCD with these symptoms. All the CCD were required to pass a blood donor health history screen and to have had a normal body temperature to donate. However, the blood donor history screen is somewhat generic, and it is possible that some donors had the somewhat non-specific symptoms of PASC, which were not captured during the health screen.

Post-acute sequelae of COVID-19 have some similarities to chronic fatigue syndrome, which is characterized by fatigue, depression, memory loss, and discomfort. Inflammatory reactions and elevated cytokine levels likely contribute to some of these symptoms. Cytokines found elevated in some chronic fatigue syndrome patients include interferon-γ, IL-6, IL-1, IL-2, and TGF-β [70]. Our study found that people who had recovered from COVID-19 were afebrile, relatively healthy but still had elevated cytokine and chemokine gene expression levels and well as increased cytokine expression throughout the 6-month study period, which suggests that immune dysregulation and immune system activation may be responsible for PASC. Consistent with our findings was another recently published report of persistent immunological dysfunction characterized by elevated proinflammatory cytokine (IFN-β, IFN-λ1, IFN-γ, CXCL9, CXCL10, IL-8, and sTIM-3) levels up to 8 months after mild-moderate COVID-19 infection. Furthermore, these were elevated in individuals with or without clinically identifiable long COVID syndrome when compared to individuals who were infected with other (non-COVID) prevalent coronaviruses or in unexposed healthy control groups [71].

## Conclusions

Overall, our study identified important gene expression trends in CCD compared to healthy donors in the post-acute period. These pathways and changes in expression levels may help inform the pathophysiology of the post-acute syndrome, not only for COVID but also for other viral diseases. Our data may serve as the basis for risk modification strategies in the period of active infection. Avenues forward will also inform potential druggable targets during convalescence from COVID-19.

## Supplementary Information

**Additional file 1: Table S1**. Genes detected by the nCounter@ Human Host Response panel.

**Additional file 2: Table S2**. Genes detected by the nCounter@ Human TCR diversity panel.

**Additional file 3: Table S3**. Expression of each gene by CCDs at 26-89 days, 90-119 days, 120-149 days and 150-241 days.

**Additional file 4: Table S4**. Comparison of the expression of VJ gene combinations among CCDs and healthy donors.

**Additional file 5: **Fig. S1. A)Total Anti-SARS-CoV-2, B)IgG Anti-SARS-CoV2, and C)Neutralizing antibody titers in individuals who donated on one or more occasions.

## Data Availability

The raw data and normalized counts are available online on Gene Expression Omnibus (GEO) in the database repository for the Nanostring host immune responses (GSE211378) and TCR diversity (GSE211394).

## Abbreviations

CCD: COVID-19 convalescent donor
HD: Healthy donors
SARS-CoV-2: Severe acute respiratory syndrome coronavirus-2
BMI: Body Mass Index
ANC: Absolute neutrophil counts
AMC: Absolute monocyte counts
ALC: Absolute lymphocyte counts
pDCs: Plasmacytoid dendritic cells
IFN: Interferon
IL: Interleukin
NK: Natural killer cells
TCR: T cell receptor
MHC: Major histocompatibility complex
CDRs: Complementarity-determining regions
PI3: Phosphatidyl-inositol-3
PLC: Phospholipase C
JAK: Janus kinase
STAT: Signal Transducers and Activators of Transcription
MAP: Mitogen-activated protein
SCGF-b: Stem Cell Growth Factor-beta
MAP: Mitogen-activated protein
HERC5: HECT and RCC1-containing protein 5
ISG: Interferon-stimulated gene
DEG: Differentially expressed genes
