# Supplementary material for: Longitudinal transcriptional analysis of peripheral blood leukocytes in COVID-19 convalescent donors
Source: J Transl Med. 2022 Dec 12;20:587. doi: 10.1186/s12967-022-03751-7 (PMC9742656; doi:10.1186/s12967-022-03751-7)
Supplement: Supplementary file 5 — Additional file 5: Fig. S1. A)Total Anti-SARS-CoV-2, B)IgG Anti-SARS-CoV2, and C)Neutralizing antibody titers in individuals who donated on one or more occasions. [file 12967_2022_3751_MOESM5_ESM.pdf]

Supplementary Figure S1

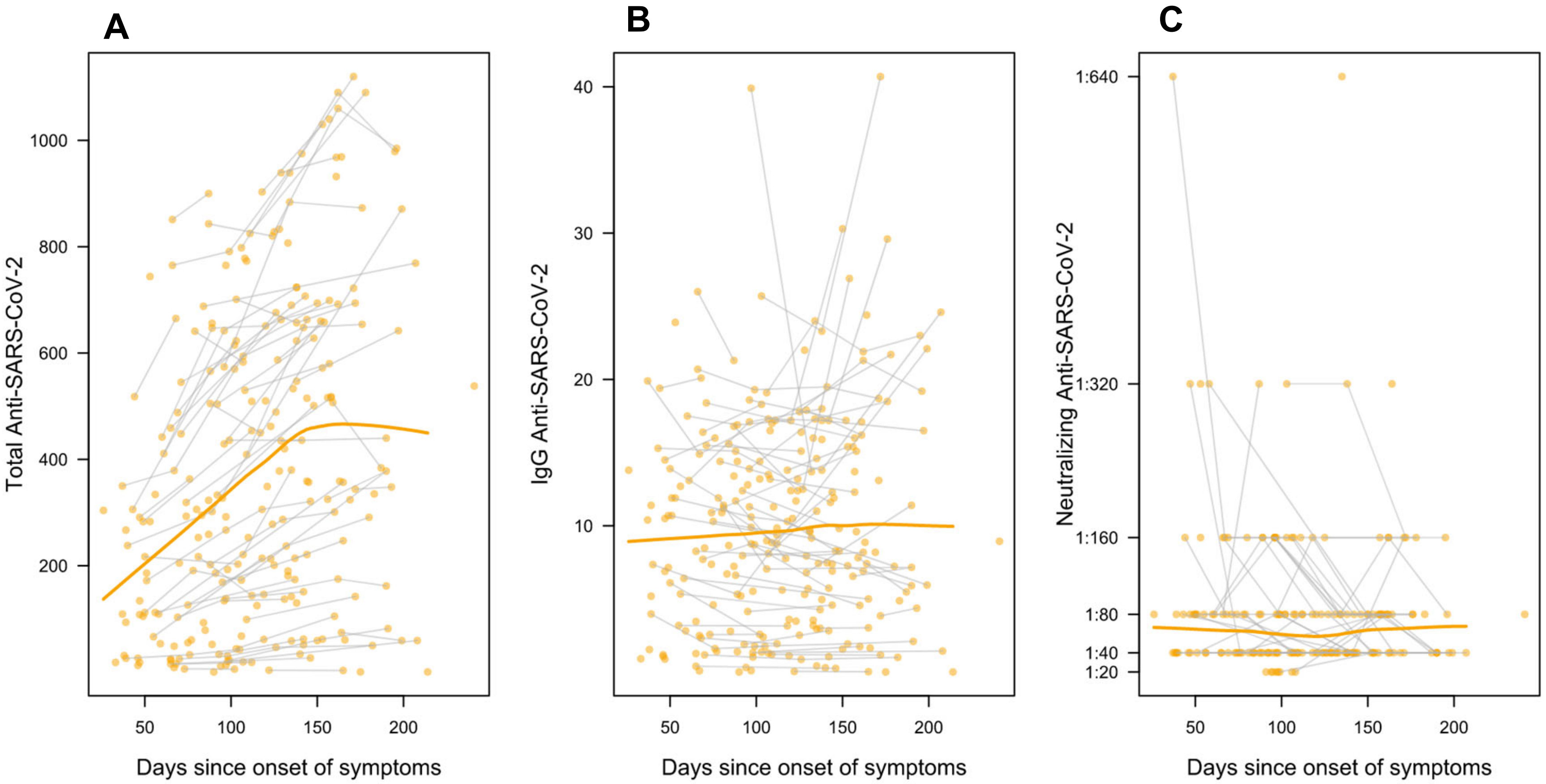

A)Total Anti-SARS-CoV-2, **B)**IgG Anti-SARS-CoV2, and **C)**Neutralizing antibody titers in individuals who donated on one or more occasions.
